# Infectious Costochondritis With Sternal Osteomyelitis

**DOI:** 10.7759/cureus.80132

**Published:** 2025-03-06

**Authors:** Koichi Fujiu, Kazuhide Uesugi, Yoshiyuki Maruya, Satoru Kayama, Hiroyuki Suzuki

**Affiliations:** 1 General Thoracic Surgery, Southern TOHOKU General Hospital, Koriyama, JPN; 2 Orthopedics, Southern TOHOKU General Hospital, Koriyama, JPN; 3 Thoracic Surgery, Fukushima Medical University, Fukushima, JPN

**Keywords:** costochondritis, osteomyelitis treatment, pseudomonas aeruginosa pathogenesis, radical debridement, sternum

## Abstract

Costochondritis is primarily caused by physical exertion, repetitive movements (such as lifting heavy objects), and severe coughing. Although it is an inflammatory condition, it is not an infection and is often treated with non-steroidal anti-inflammatory drugs (NSAIDs). In contrast, infectious costochondritis usually develops when an infection spreads directly from a postoperative wound or adjacent foci. We present a case of infectious costochondritis with sternal osteomyelitis caused by *Pseudomonas aeruginosa*, where the infection did not spread from adjacent tissues. A 59-year-old man was referred to our hospital with anterior chest pain and swelling persisting for three months. He had been diagnosed with diabetes mellitus three years prior. Three weeks before his visit, a purulent exudate had fistulized into the skin. Two weeks prior, he had sought care from a nearby doctor, who diagnosed a subcutaneous abscess and performed an incision and drainage. Cultures identified *P. aeruginosa*. However, the condition did not improve, and chest computed tomography (CT) was performed, showing edema around the seventh costal cartilage, the inferior end of the sternum, and surrounding subcutaneous tissue. Distraction of the seventh costal cartilage was also noted. Magnetic resonance imaging (MRI) with fat-suppressed T2-weighted images showed high intensity in the same area. Blood cultures were negative. Based on these findings, we diagnosed costochondritis and sternal osteomyelitis. Treatment began with oral cefalexin (CEX) for seven days, followed by oral cefcapene pivoxil hydrochloride hydrate (CFPN-PI) for 14 days. During hospitalization, meropenem hydrate (MEPM) was administered. After seven days of MEPM, the seventh costal cartilage and part of the sternum were debrided under general anesthesia. Indicators of the extent of debridement included preoperative MRI, bone cortex hardness under intraoperative palpation, and bone bleeding. MEPM was administered for 14 days, including preoperative treatment, followed by cefepime dihydrochloride hydrate (CFPM) for 14 days and levofloxacin hydrate (LVFX) for seven days. After 11 months of follow-up, there was no recurrence of costochondritis or osteomyelitis. Infectious costochondritis with sternal osteomyelitis caused by *P. aeruginosa* was successfully treated with debridement. Properly determining the extent of debridement perioperatively is crucial for effective treatment.

## Introduction

Costochondritis is primarily caused by physical exertion, repetitive movements (such as lifting heavy objects), and severe coughing. It typically affects the costochondral junctions of the second to fifth ribs in individuals aged 40-50 years old and should be differentiated from ischemic heart disease. Although it is an inflammatory condition, it is not an infection and is often treated with non-steroidal anti-inflammatory drugs (NSAIDs) [1].

In contrast, infectious costochondritis usually occurs when the infection spreads directly from a postoperative wound or adjacent foci [2,3]. Common causative organisms include *Candida*, *Salmonella enterica* serotype Choleraesuis, *Escherichia coli*, and *Staphylococcus aureus* [4]. The condition has been reported in immunocompromised individuals or drug users [5].

Sternal osteomyelitis is commonly caused by median sternotomy, chest trauma, or irradiation, resulting from direct transmission of infection from surrounding infected tissues. There are also rare cases of hematogenous spread [6].

In this study, we report a case of infectious costochondritis caused by *Pseudomonas aeruginosa*, accompanied by adjacent sternal osteomyelitis.

## Case presentation

This case describes a 59-year-old man who underwent lumbar disc herniation surgery 15 years ago. Eight years prior, he experienced a transient ischemic attack and began taking clopidogrel 75 mg/day. Three years ago, he was diagnosed with diabetes mellitus and started sitagliptin 50 mg/day. His blood pressure was 122/80 mmHg, and he was also taking azilsartan 20 mg and amlodipine 5 mg. Additionally, he had hyperlipidemia and was on rosuvastatin 5 mg/day. He had a 39-pack-year smoking history until hospitalization and worked in the restaurant industry.

Three months prior to the presentation, the patient developed anterior chest pain with coughing. Three weeks before admission, a purulent exudate fistulized into the skin. Two weeks before visiting our hospital, he visited a nearby doctor and underwent incision, pus drainage, and wound cleaning after being diagnosed with a subcutaneous abscess. He was treated with oral cefalexin (CEX) 1,000 mg/day for seven days, followed by oral cefcapene pivoxil hydrochloride hydrate (CFPN-PI) 300 mg/day for 14 days. Cultures identified *Pseudomonas aeruginosa*. However, there was no improvement. Chest computed tomography (CT) was performed, revealing costochondritis with sternal osteomyelitis, and the patient was referred to our hospital.

**Figure 1:**
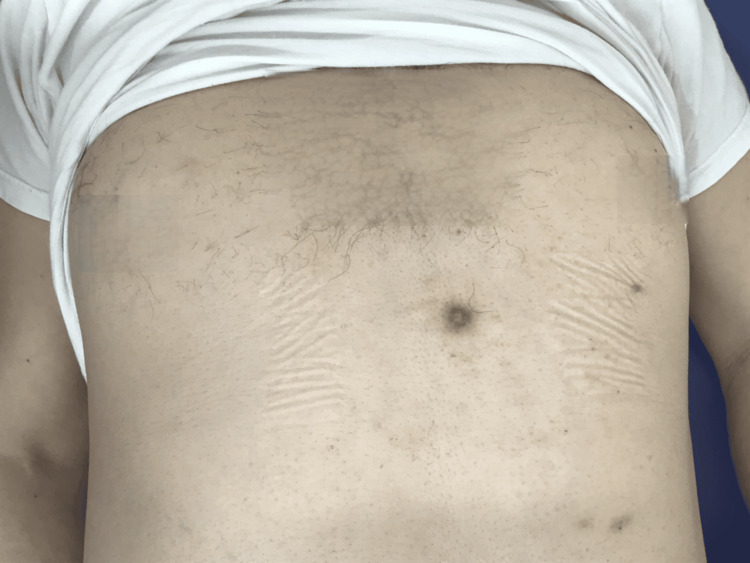
Anterior thoracic appearance Pustules are present on the left anterior chest.

**Figure 2:**
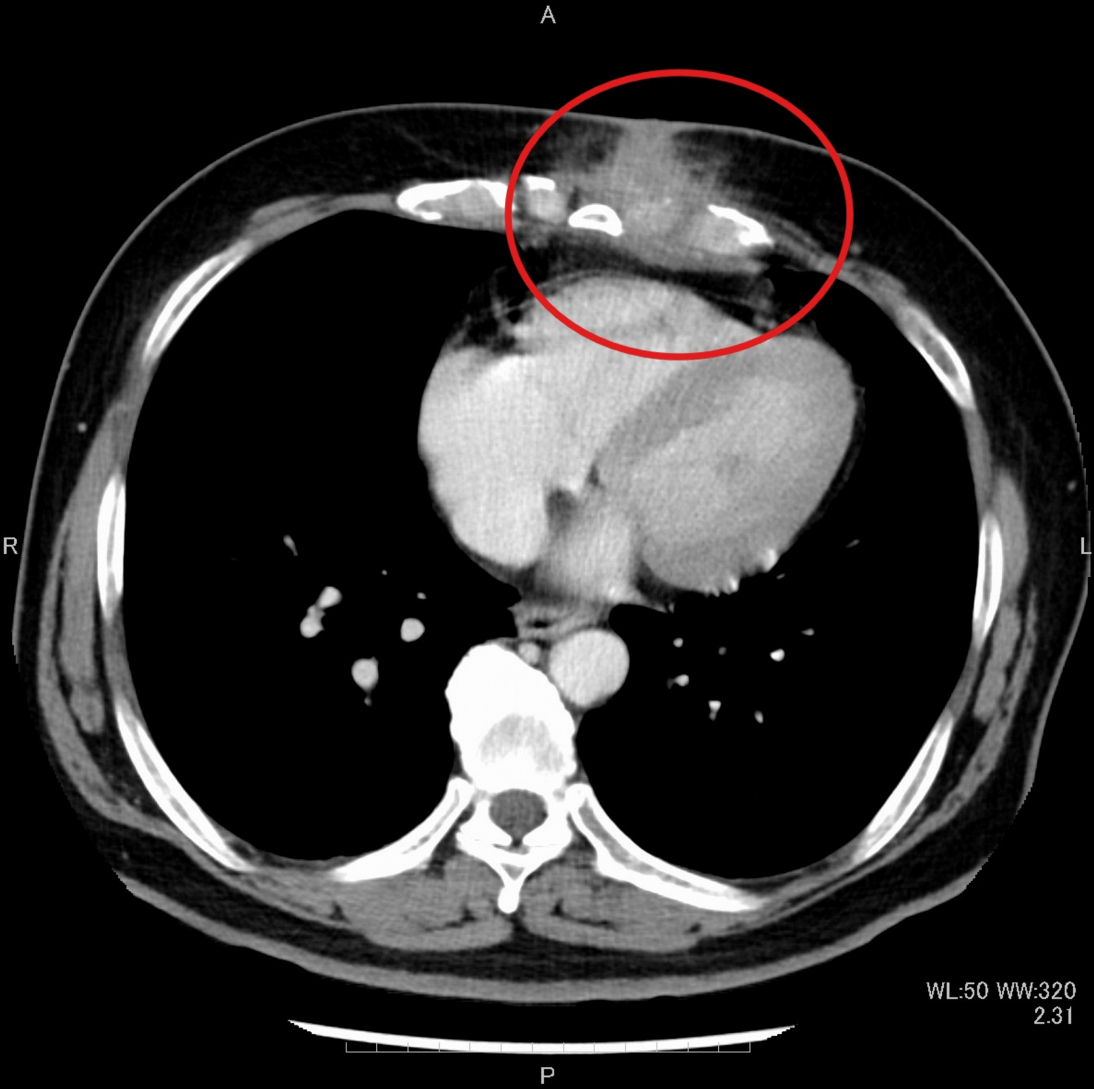
Computed tomography showing a high-density area around the inferior end of the sternum and surrounding subcutaneous tissue. The seventh costal cartilage is distracted (red circle)

**Figure 3:**
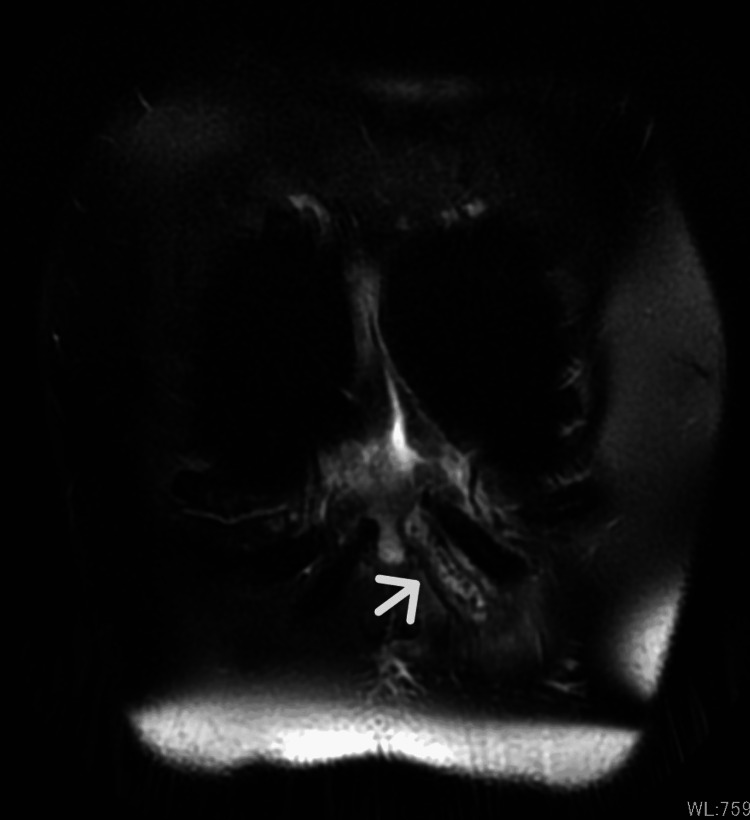
Magnetic resonance imaging with fat-suppressed T2-weighted image showing scattered high-intensity signals in the seventh costal cartilage (arrow)

**Figure 4:**
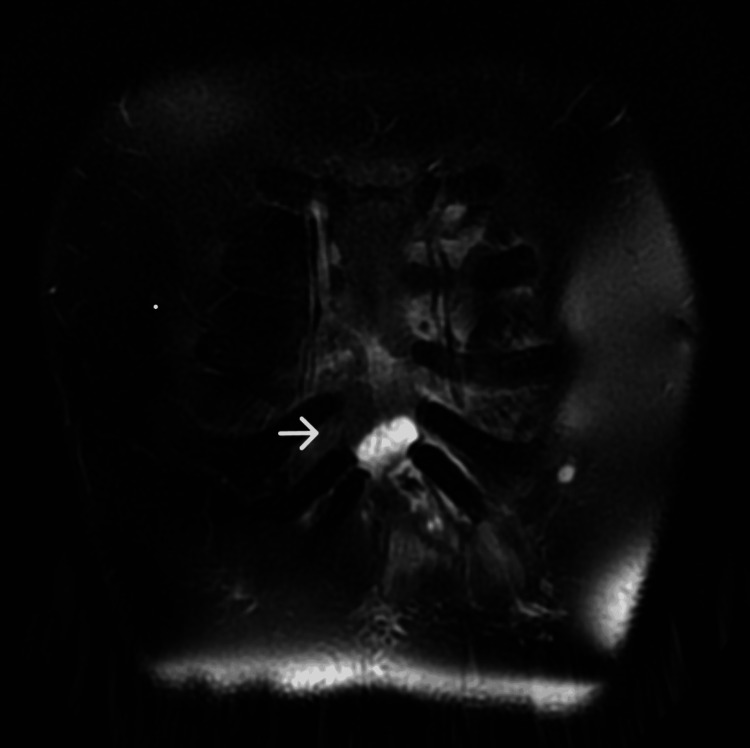
Magnetic resonance imaging with fat-suppressed T2-weighted image showing high intensity in the inferior sternum (arrow)

**Figure 5:**
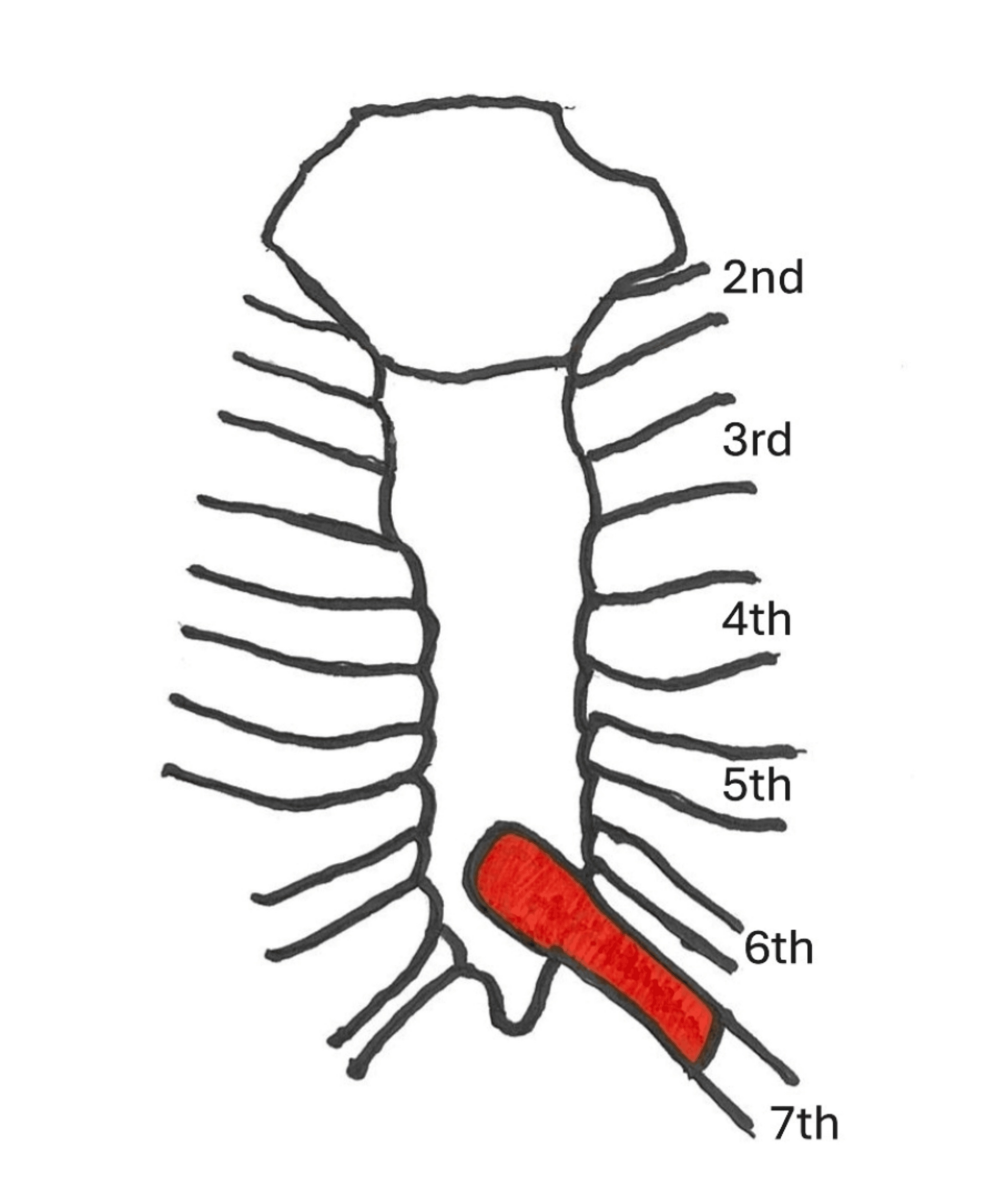
Operative findings showing the area that has been debrided (red fill) Image Credit: This image was recreated by the authors based on the CT and MRI scans of this case.

**Figure 6:**
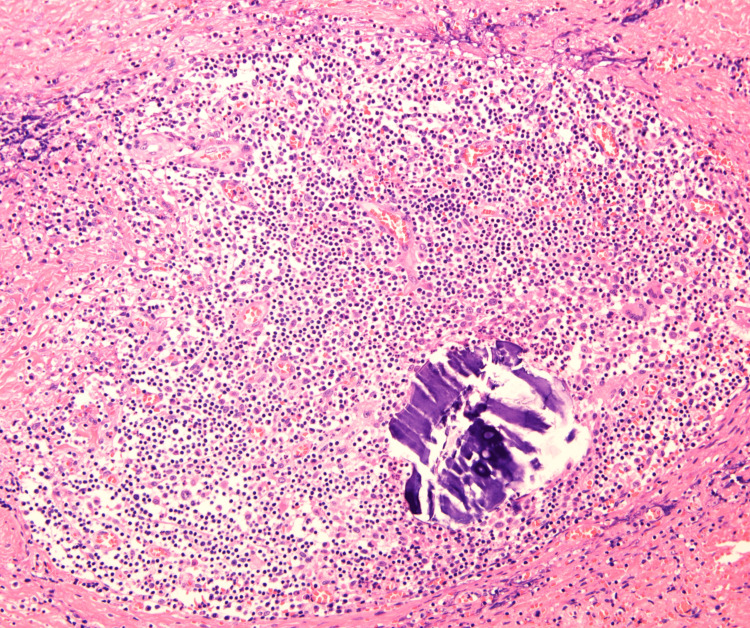
Histopathology of the debrided tissue In the debrided tissues, focal inflammatory cell infiltration centered on plasma cells is observed microscopically (hematoxylin and eosin stain at 200x magnification).

**Figure 7:**
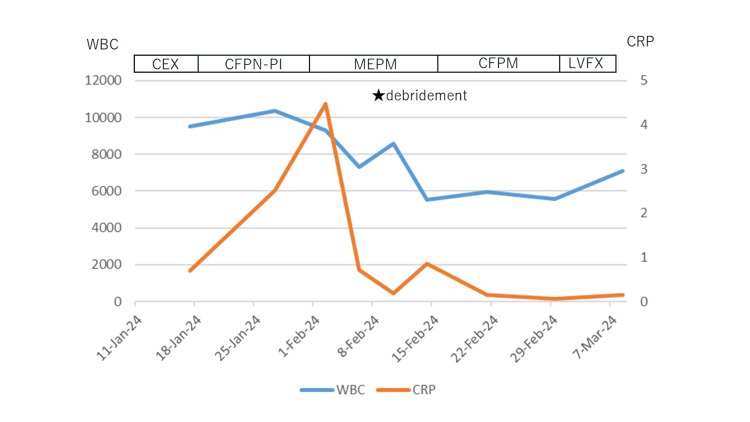
Clinical course of the patient CEX: cefalexin, CFPN-PI: cefcapene pivoxil hydrochloride hydrate, MEPM: meropenem hydrate, CFPM: cefepime dihydrochloride hydrate, LVFX: levofloxacin hydrate.

Upon initial diagnosis, the patient had no fever, with a white blood cell (WBC) count of 10,380/μL, C-reactive protein (CRP) level of 2.51 mg/dL, and hemoglobin A 1c (HbA1c) of 6.6%. Pustules were also observed (Figure 1). Blood cultures were negative. Chest CT revealed edema due to inflammation around the seventh costal cartilage, the inferior end of the sternum, and surrounding subcutaneous tissue. Distraction of the seventh costal cartilage was noted (Figure 2). Magnetic resonance imaging (MRI) with fat-suppressed T2-weighted images showed scattered high intensity in the seventh costal cartilage (Figure 3) and inferior sternum (Figure 4). An echocardiogram showed no signs of endocarditis. Following hospitalization, the infected costal cartilage and sternum were debrided after the administration of meropenem hydrate (MEPM) 3 g/day for one week. The seventh costal cartilage and inferior sternum were debrided under general anesthesia through an oblique incision in the left lower anterior chest. A sharp spoon was used for debridement. Indicators of the extent of debridement included preoperative MRI, bone cortex hardness under intraoperative palpation, and bone bleeding. The bone cortex on the mediastinal side of the sternum was preserved as it required no debridement. Additionally, since the costal cartilage is avascular and vulnerable to infection, more than 2 cm from the infection site was removed, as indicated by the preoperative MRI. Moreover, the non-infected fifth and sixth sternocostal articulations were spared (Figure 5). A subcutaneous drain was placed and removed on postoperative day 6. Histopathological examination of the debrided tissues revealed focal plasma cell-based inflammatory infiltration and granulation tissue (Figure 6). MEPM was administered for 14 days, including the preoperative treatment, followed by cefepime dihydrochloride hydrate (CFPM) 4 g/day for 14 days and levofloxacin hydrate (LVFX) 500 mg/day for seven days. After 11 months, there was no recurrence of costochondritis or osteomyelitis. The patient's clinical course is shown in Figure 7.

## Discussion

The cause of infectious costochondritis is often a contiguous infection from adjacent purulent foci, such as trauma, cellulitis, or subcutaneous abscesses [2,3]. Additionally, bacteria from other body parts have been reported to infect costal cartilage in a homogeneous manner [7].

In our case, there was no focus on infection in adjacent organs or other body parts, and blood cultures were negative. The pustules on the anterior chest are unlikely to be the source of the infection, as they appeared three months after the onset of pain and swelling. The infection of the left seventh costal cartilage was most pronounced, and the sternal osteomyelitis was confined to the area adjacent to the left seventh costal cartilage. Therefore, it is believed that the infectious costochondritis progressed to the sternum, causing sternal osteomyelitis. Although diabetes mellitus may have been a contributing factor, the source of the *P. aeruginosa* infection in the costal cartilage remains unknown.

MRI is effective in assessing the extent of costochondritis and osteomyelitis in the adjacent sternum [3].

In this case, the patient had been taking oral antibiotics for three weeks before referral to our hospital. After hospitalization, antibiotics were administered for an additional five weeks. For vertebral osteomyelitis, the recommended duration of antibiotic treatment is six weeks [8]. The duration of antibiotic therapy depends on factors such as the presence of debridement, clinical course, inflammatory markers, culture results, and imaging findings [9] and is not predetermined.

The extent of costal cartilage debridement is crucial, as the cartilage is vulnerable to infections due to its avascular nature, relying on diffusion from surrounding tissues for oxygen and nutrients. Consequently, more than 2 cm of the surrounding tissue was removed from the exposed part of the costal cartilage [6]. In cases of recurrence, debridement up to the costochondral junction may be necessary [10].

## Conclusions

Infectious costochondritis with sternal osteomyelitis caused by *P. aeruginosa* was successfully treated with debridement. The source of the infection remains unknown. MRI is effective in assessing the extent of costochondritis and the extent of osteomyelitis in the adjacent sternum. The extent of debridement should be carefully determined perioperatively.
